# Living Matter(s) for Learning: An International, Multi-Sited Ethnography Exploring How Surgeons’ Learning Is Mediated Through the Use of Live Animal Simulation

**DOI:** 10.5334/pme.1762

**Published:** 2025-04-23

**Authors:** Cara Swain, Charlotte Silén, Klas Karlgren

**Affiliations:** 1 Department of LIME, Karolinska Institute, SE; 2 Academic Department of Military Surgery & Trauma, Royal Centre for Defence Medicine, UK; 3 Department of LIME, Karolinska Institutet, SE; 4 Department of Research, Education, Development and Innovation, Södersjukhuset, SE; 5 Faculty of Health and Social Sciences, Western Norway University of Applied Sciences, NO

## Abstract

**Introduction:**

Surgical simulation training substituting a live animal for a human patient is a continuing practice. Despite clear ethical controversy, many perceive this type of simulation to be ‘high fidelity’ and therefore valuable. This study employs a sociomaterial perspective to explore how use of a live animal mediates learning activity and behaviour during a trauma surgical simulation course.

**Methods:**

This international, focused ethnography generated data through observation of surgical simulation courses in six different countries. A narrative analysis was conducted using instrument-mediated learning theory as a lens for interpretation.

**Results:**

The key finding is the dual and fluid existence of a live animal as an instrument for learning, variably perceived as a simulator tool for training and as a patient that must be saved. When framed as a tool, surgical knowledge and skills are practiced with learning acquired via epistemic and pragmatic mediation. Performing a thoracotomy denotes a critical moment; procedural unfamiliarity, evident haemorrhage and inherent risk of a deadly outcome contribute to uncertainty and clinical complexity. Learners are hence more likely to frame the animal as a patient. This experience has psychological fidelity, feeling more authentic as actions have consequences. Risk of failure to sustain the life of the animal mediates reflexive learning, teaching the learners about themselves and their abilities.

**Conclusion:**

Live animal simulation training mediates surgical learning differently, dependent on whether the animal is framed as an instrument or as a patient. The animal’s ability to bleed and exsanguinate to death creates risk and uncertainty as learners perform complex skills under pressure of significant consequence. Authenticity could be amplified if the animal is framed as a patient throughout the simulated learning event.

## Introduction

Simulation is an active educational technique that replaces real experiences with guided ones, replicating aspects of the real world [1]. Surgical simulation training using a live animal as a substitute for a human patient, so-called “live tissue training” (LTT), is a continuing practice by surgical specialties [2,3] and other providers of trauma care [4], despite ethical concerns [5,6]. Notwithstanding advances in simulator technologies, LTT is felt to provide a learning experience that is unique or irreplaceable [7,8].

Simulation fidelity is a complex and debated topic in the medical literature [9,10,11] comprising domains of varying nomenclature [9,10,11,12,13,14,15]. Most commonly discussed are physical fidelity (how a simulator looks and feels) and functional fidelity (how a simulator behaves or what it does when interacted with). The notion of higher fidelity simulations being more beneficial for learning has been debunked [16], yet the fidelity of using a live animal is a key aspect of the LTT debate. Simply, critics argue there is lower physical fidelity due to anatomical dissimilarity, but supporters state that higher functional fidelity apparent through the presence of bleeding and a reactive physiology supersedes differences in anatomy. The concept of psychological fidelity, meaning the simulation generates cognitive, behavioural and emotional responses expected in the equivalent real-life situation [9,15] may be more relevant, with some evidence that psychological fidelity may be a more critical determinant of learning [16].

Developments in simulation-based education have typically focused on mastery of procedural skills. Research has been directed at technologies within higher-fidelity environments rather than pedagogy that can maximise how they are used [17]. Prior research has focused on comparing training using live animals to alternative simulator models [18,19] or attempted to demonstrate efficacy [20,21,22,23,24]. Some attempts have been made to explore learning beyond technical skills, but no research has explored how learning is specifically attributable to use of a live animal. Knowledge about learning processes and how LTT is used to educate surgeons is critical to contribute to a potential justification argument, which addresses the educational attributes and ethical concerns of the practice. Our purpose was to describe the learning practices and experiences that occur during a LTT course. Using sociomateriality and mediation theory as a lens for analysis, we explore: *How does the use of a live animal during a trauma surgical simulation course influence and mediate learning activity and behaviour?*

## Theoretical Framework

Learning is an experiential, dynamic, interactional process, situated in a physical, social and cultural context. Knowledge is constructed both individually and in collaboration, mediated by various tools and technologies [25,26,27]. Materials can support learning in different ways, but are often ignored as part of the background for human action [25]. A sociomaterial perspective views tools and technologies to be dynamic and enmeshed with human social activity [17,25]. Therefore, fidelity is not a fixed characteristic of a simulation, but results from interactional forces between the context, learners and the material world [17,28]. Previous research on interactional relations has focused on human patient mannequins [28,29,30], surgical part task trainers [31], computer-based [28] and virtual reality simulation [32] and human cadavers [33,34].

Instrument-mediated activity was described by Rabardel [35], informed by Vygotsky [36]. Mediated action is socially situated and constructed, with individuals granting the instrument the status as a resource for the action [35,37]. Mediation can be subject-object, directed toward development of knowledge and competencies through use of the instrument, or subject-subject, where the learner develops understanding of others, in relation to the instrument. Various mediations can be jointly present in an instrumented activity:

- Epistemic mediation – creating or working with knowledge, understanding an object and its intrinsic properties or characteristics
- Pragmatic mediation – organising, planning and coordinating knowledge-creating processes through actions on the object
- Reflexive mediation – making knowledge practices visible, reflecting on and transforming one’s own practices
- Social or interpersonal mediation – building and managing networking communities and social relations required to advance knowledge [37]

**Figure 1:**
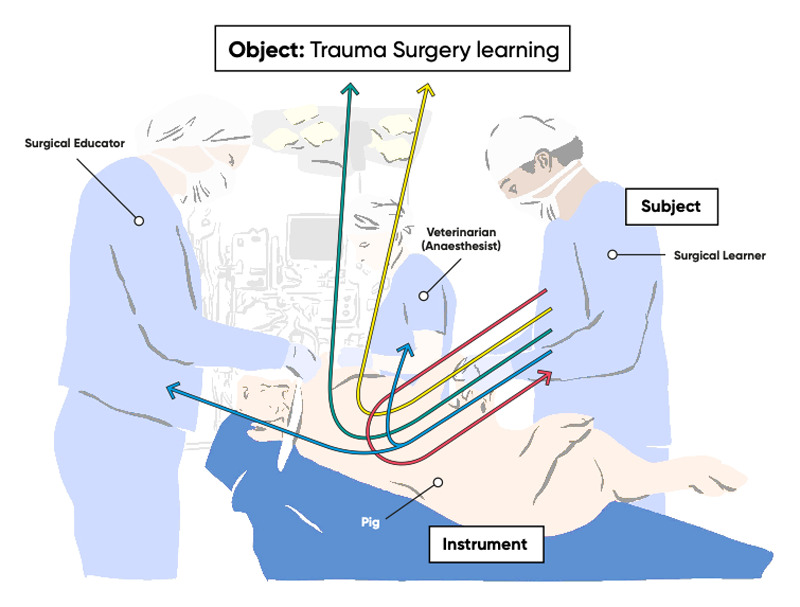
Rabardel’s theoretical description of instrument-mediated learning, applied to the context of live tissue training.

Mediation theory has previously been applied to technologies used in learning such as computer systems and virtual environments [38]. In LTT, a live animal is appropriated in place of a human patient, as an instrument to mediate learning (Figure 1).

## Methods

Our sociomaterial perspective is built on a foundation of critical realism. This philosophical position is realist, but subjectivist, assumes that an ontological material reality exists, but that epistemological knowledge about it is gained through and mediated by human senses, language, and culture.

This study was an international, multi-sited, focused ethnography [39,40] of simulation courses that used a live animal to teach surgical management of trauma. Ethnography has been used to explore surgical practice and culture [41,42,43,44,45,46] and to a lesser extent, surgical simulation [32,47]. Focused ethnography is a legitimate, well-suited method of exploring specific episodes in social fields and a useful choice for researchers with a clear frame of inquiry and prior cultural familiarity [39,40].

### Setting and participants

Simulation courses were observed in Canada, USA, France, Norway, Germany and Sweden during 2023 (Table 1). The physical locations varied between bespoke clinical settings designed for training using animals, or housed within a hospital complex such as a repurposed basement storage facility or former anatomy laboratory. Access to all locations where LTT took place was strictly controlled, with presence of animals and overall purpose of the site not advertised.

**Table 1:** Description of observed courses and characteristics.


LOCATION	LEARNER DEMOGRAPHIC	TOTAL COURSE DURATION (DAYS)	OBSERVED DURATION (HOURS)	OBSERVED LTT (HOURS)	LEARNER: EDUCATOR RATIO PER ANIMAL

Canada	Surgeons	3	26	3	4 : 1

France	Surgeons	3	5	4	3–4 : 0.5

Germany	Surgical teams	6	33	6	4 : 2

Norway	Surgical teams	5	42.5	22	6 : 2

Sweden (1)	Pre-hospital trauma teams	3	11	7	3–4 : 2

Sweden (2)	Surgical teams	3	23.5	7.5	4 : 1

USA	Surgeons	1	8	3	2 : 1

		Total	149	52.5	


The principal learners were typically surgeons of different specialties; surgical team training also featured anaesthetists and other emergency allied health professionals, including paramedics and nursing staff. At two locations, live animals were the only patient simulators used. Typically multiple modalities were used for training, usually human cadavers or part-task trainers. None of the courses used a live animal on more than one day. Faculty conducting live animal training were surgeons, however other members of faculty were often from multiple disciplines, reflecting the composition of the learner cohort. Veterinary specialists and additional staff were used to coordinate the simulation and manage the logistical elements of live animal training.

### Data generation and analysis

CSw attended seven trauma simulation courses, in the role of observer between March to November 2023 for a total of 149 hours, of which 52 hours were LTT. Ethnographic observation [48] was the primary source of data. Preliminary notes were written during the observation sessions to capture key details and initial analytic thoughts about learners’ and educators’ activities. Documents and other media associated with the courses, such as presentations, instructions provided to the learners, or faculty handbooks were also reviewed. Raw field data from each site were transcribed into diachronic ‘thick’ [49] descriptive electronic fieldnotes daily and reviewed on completion of the observation period at each host site [50]. The complete fieldnote corpus comprised 79 pages.

Alongside ethnographic data, semi-structured interviews were conducted. Faculty educators at all courses were invited to participate in a virtual interview, with learners from three courses permitted by the host institution to be recruited. Three learners and four educators accepted, received additional study information, and signed a written consent form in advance. Interviews were conversational in nature, using a pre-written guide which was tailored to the participant’s role and based on observed activities. Recorded data was transcribed verbatim.

Narrative analysis was guided by the process of narrative methodology [51]. It was an iterative process with initial data generation guiding future observations and interview topics [48]. Fieldnotes were shared and significant events – notable situations that raised questions, elicited strong emotions or focused on a topic of interest [52] – presented for discussion to the research team. These events were considered as part of a broader whole to develop plots – narratives that meaningfully organised characters, events, actions and intentions [51,52] – characterising the learner’s evolving experience during LTT. Abductive interpretation of these events and relevant educational theories, latterly mediated learning theory, formed our answer to the research question.

### Team composition and Reflexivity

CSw, as lead researcher, held a dual insider-outsider position throughout the study. She is employed by the British Armed Forces and works clinically as a surgical registrar/resident. Being familiar with the knowledge practices, principles, environment and language of trauma surgery facilitated contextual immersion and guided the initial analysis. Keeping reflexive notes [48] throughout, including regarding how the research study and her presence as a researcher affected social dynamics and situations was beneficial, and regularly discussed.

CSi has a background in clinical nursing, and significant experience with healthcare education, faculty development and an academic focus on medical education research. KK is an educational researcher with academic interest in simulation and use of technologies for learning. Both provided alternative perspectives to the generated data and encouraged complementary reflection on observed situations. We all hold a neutral stance regarding the use of live animals (considering ourselves neither for or against LTT), and agree with the principles of the 3Rs of humane animal research and experimentation [53].

### Ethical considerations

This research study was validated by the Swedish Ethical Review Authority [Dnr 2022-01884-01]. Learners and faculty were informed of the presence of a researcher and provided with written information about the study in advance. Detailed descriptions of the courses have deliberately not been reported to preserve anonymity of the locations and persons involved in the training.

## Results

**Figure 2:**
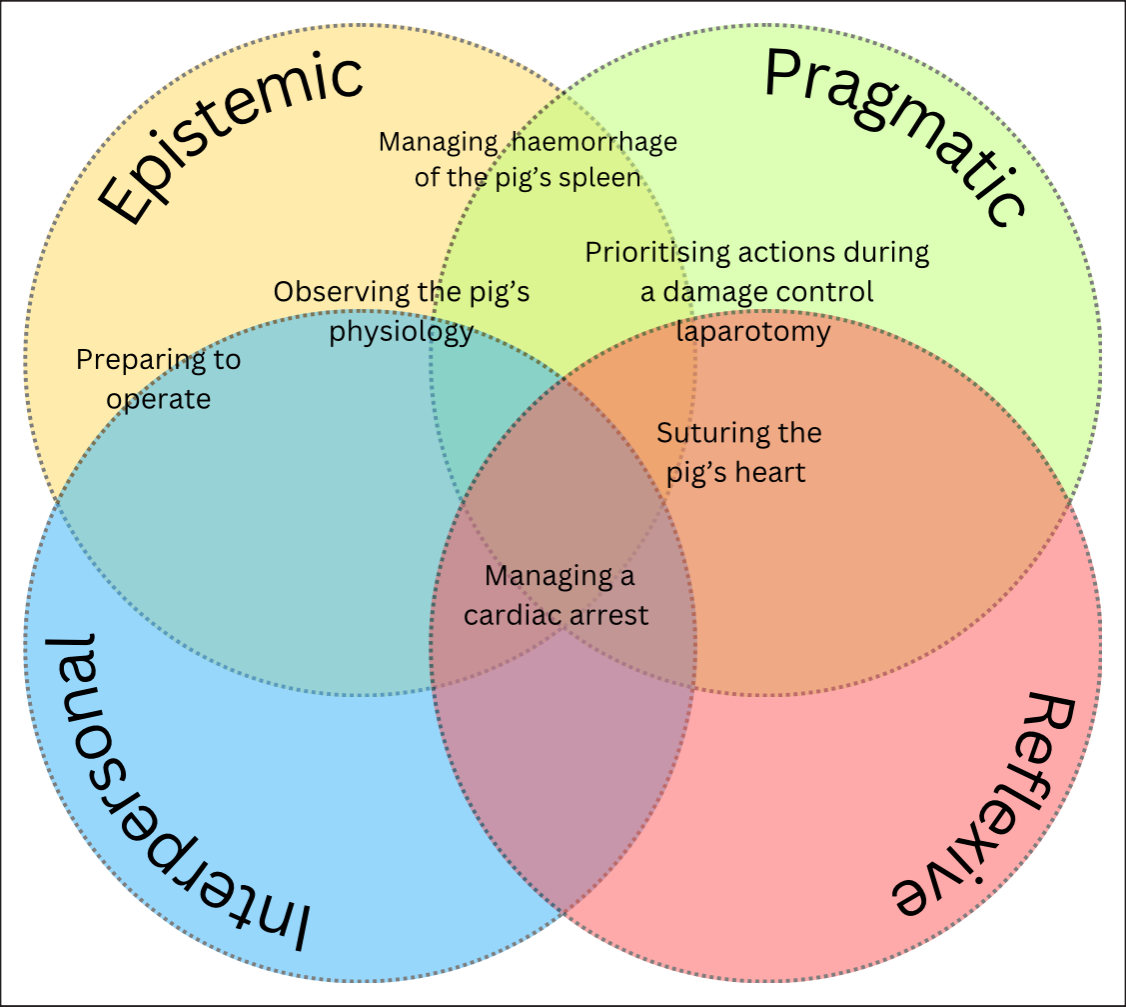
Diagram mapping learning events to types of mediation using a live animal.

Narrative vignettes are used to portray significant events through a surgical learning experience and support one interpretation of how live animal use mediates learning; additional interpretation is presented in Table 2 and Figure 2. Vignettes are composited from various similarly-observed events, and should not be considered as descriptions of any one specific event.

**Table 2:** Examples of events occurring during LTT and how learning may be mediated.


VIGNETTE	SUMMARY OF EVENT	KEY DESCRIPTIVE FEATURE	TYPE OF MEDIATION

Setting the scene	Preparing to operate	The learner uses their understanding and interpretation of the environment to choose a pig to operate on	Epistemic

		The learner recognises that they are not able to start the simulated surgery until the veterinary staff are satisfied that it is appropriate	Interpersonal

Controlled professional skills development	Managing haemorrhage of the pig’s spleen	The learner recognises significant bleeding from the spleen and understands this must be controlled	Epistemic

		The learners interact with the pig’s spleen to develop a plan of how the haemorrhage can be controlled	Pragmatic

	Prioritising actions during a damage control laparotomy	The learners make a choice to sequence their actions, suturing the bowel before looking for sources of bleeding in the abdomen	Pragmatic

		The learners recognise that their understanding of the principles of trauma surgery were incorrect when presented with a clot adherent to the mesentery	Epistemic

The heart of the matter	Suturing the pig’s heart	The learners interact with the pig’s heart to develop a plan of how the haemorrhage can be controlled by placing a suture	Pragmatic

		The learners recognise their own limitations in terms of ability and adapt for subsequent attempts	Reflexive

	Observing the pig’s physiology	The learner recognises that the fibrillation of the heart correlated with the displayed abnormal is not normal	Epistemic

		The learner interacts with the veterinarian to learn that the pig is in distress and the abnormal physiology is a cause for concern	Interpersonal

Success and failure, life and death	Managing a cardiac arrest	The learners work together and interact with the veterinary support staff to perform as a team to resuscitate the pig	Interpersonal

		The learners manage their own stresses and emotions to perform their tasks under pressure	Reflexive

		The learners recognise the requirement to start resuscitative actions in response to significant cardiac bleeding	Epistemic

		The learners interact with the pig’s heart and sequence their actions to appropriately and safely resuscitate the pig	Pragmatic


### Setting the simulated scene

*The pigs are pre-positioned on the operating tables, lying on their backs with their trotters tied to the metal frame to fully expose their bellies. Each has one ear bandaged to keep the cannula in place that is providing their medication. There is another plastic pipe protruding from the skin in their neck and the breathing tubes displace their tongues from their mouths. The anaesthetic machine next to each pig whirs and hums as their chests gently rise and fall. The adjacent screen depicts the usual parameters of ventilation, heart rate, and blood pressure as a series of moving coloured lines and numbers*.

*The surgical learners, now wearing their familiar outfits of scrubs, operating gowns, gloves and masks are taking in the scene, moving between the tables. There is an air of excitement. One learner is looking at all of the monitors in turn, explaining “I want the best looking pig”. Content with the numbers observed on the screen, the learner stands waiting beside one of the operating tables as a member of the veterinary staff uses a pair of metal tongs to firmly squeeze the trotter of one of the pigs. Satisfied that the pig is suitably anaesthetised, they write a note on the chart next to the operating table and indicate that this team can start*.

*Within minutes, the learners have performed a laparotomy and the internal organs of the pigs are exposed. The monitors are now ignored and the familiar sounds of operative practice become background noise as the educators begin to teach. On the wall behind each operating table is a sign with a list of procedures to be practiced suffixed with a statement proclaiming ‘DEATH IS NOT AN ENDPOINT’*.

The physical fidelity of the operating theatre can be considered more or less realistic; there is working equipment for anaesthesia and physiological monitoring and a safe clinical context to allow for management of blood and other fluid spillage. Familiarity of surgical practice is present due to a series of sensory events. Learners and faculty dress to operate and enter the scene with their ‘patients’ already anaesthetised and positioned. Each pig has an observation chart recording their vital signs and dosages of administered drugs. Many are cared for on a 1:1 basis and routinely checked for signs that would indicate inadequate anaesthesia, although the frequency of these observations varies. These activities, the sounds of the equipment, the feel of the tissues and the smells produced once operating through use of the pig evoke known memories for surgeons.

However, disruptions in the environmental and functional fidelity of the operative performance clearly indicate that LTT is not a human patient clinical experience. There is decreased consideration regarding sterility of equipment (*i.e*. instruments falling to the floor, reuse of a dropped cannula) and snacks and beverages are provided within the operating theatre for learners and educators. There is limited interaction between the learners and educators who were training and the veterinary staff caring for the pigs, contrary to the significant and necessary communication between surgeon and anaesthetist that occurs during human trauma cases. Notably, there is no interaction required with the ‘patient’ in advance and no consent process. The fact that the pigs are unwilling participants is little discussed, due to an understanding among those observed that this type of learning is necessary.

The signs displaying the curriculum content alongside the euthanasia procedure and statements such as *‘Bleeding control ++ The pig must stay alive until the end of the procedure!’* establish early the serious expectation of what is required from the learners, but also indicate the dual ontology of the animal, as a model for learning about haemorrhage control, and a patient that must be cared for… at least until the end of training.

### Controlled professional skill development

The focus during LTT is the acquisition of complex psychomotor skills, and being able to use knowledge about trauma surgery principles to create and enact a management plan, to stop bleeding and treat injuries in an acute surgical emergency. Educators create injuries which progress from minor to more significant, resulting in major haemorrhage that must be controlled. Learners demonstrate their ability to perform a certain task on the animal, with the practice of decision-making, learning to deal with complexity and managing their individual stress levels, secondary goals or perhaps unanticipated consequences.

*The educator tells all the learners to step away from the operating table and turn their backs to obscure their view. He and the scrub nurse lean forward and he lifts the spleen from the open abdomen. A pig’s spleen doesn’t look like a human spleen – it has a flat, curved oblong appearance, like a tongue, and is very mobile, unlike the fist-sized organ fixed in the left upper quadrant of a person. Using scissors, the educator makes a cut into the tissue. There isn’t much bleeding, so he cuts again, this time through a vessel – “yeah you got it” the scrub nurse murmurs as the blood spurts out. The educator uses his thumb and forefinger to stem the flow and turns to the learners. “Stab wound to the left flank. You’re going to have to move fast.” As they return to the operating table, unclasping their gloved hands, he drops the spleen into the abdomen and quickly covers it with the pig’s intestines before moving aside to allow the operating surgeon to get his hands in to work*.

*At another table, another educator peers over the shoulders of two of the learners. They are carefully suturing a laceration in the small bowel. “What are you doing?” He seems displeased. “What was the scenario?” They reply that it was multiple stab wounds to the abdomen. He asks if there was blood in the abdomen when they ‘went in’. Yes. He asks why they have wasted time fixing a bowel injury and not looking for the source of bleeding? “That is an error” he states, pushing between the learners to reach into the pig’s abdomen and lift the bowel into view. There is blood pooling in the mesentery with a clot adherent to the tissue. The educator reiterates that identification and management of bleeding is paramount in trauma surgery; bowel contamination can be dealt with later*.

The animal is instrumentalised as a model for surgical practice with predominant pragmatic mediation. At the first operating table in this vignette, the animal is used to demonstrate significant bleeding, allowing the learners to apply their knowledge of how to manage haemorrhage. A simple scenario prefixes the required task and gives context to the learning aim: quickly find and stop the bleeding. It doesn’t matter that the pig’s spleen looks different (lower physical fidelity), as all the surgeons know what a human spleen looks like, but the functional fidelity is paramount; the simulated organ behaves and responds in the expected manner, as the learner performs actions to control the bleeding.

Later in the vignette, there is further evidence of epistemic with pragmatic mediation. Learners can apply their knowledge of trauma principles and practically interact with the animal, but in this instance their applied knowledge and decision-making is clarified by the educator to be flawed. The presence of clotted blood within the bowel mesentery demonstrates their delay in managing haemorrhage while inappropriately prioritising stitching the intestine.

Absent is any response to, or interaction with, the presented physiology. This was variable throughout the different courses, but typically does not feature during this early training stage. The physiological parameters are used by the veterinary staff only to monitor the animals. At times, educators can be seen observing the numbers, possibly to make judgements about which procedures to perform next or how successful the learners are being in managing blood loss. Alarms signalling adverse physiology are ignored or silenced, perhaps to prevent the learners being distracted or potentially because the information about the animal’s physiological status is considered unimportant while instrumentalised as a model.

### The heart of the matter

Performing a thoracotomy in clinical practice is rare, unlike a laparotomy, which most surgeons will perform multiple times during their training. One course director stated only 5% of chest injuries lead to massive haemorrhage or cardiac wounds, clarifying that was “why we’re here today” to practice skills and “get extra reps in”. LTT is an opportunity to create knowledge for those with no experience, or apply their limited experience and transfer skills to a rare situation.

Performing the thoracotomy indicated the culmination of training and the end of the animal’s life. When the time came to make that incision and open the chest of the pig, it was always associated with a noticeable, palpable change in the atmosphere. Increasing noise and chaotic activity signalled the start of a surgical learning activity involving serious major bleeding, and requiring critical action from all involved. Learners’ emotions became more apparent, the most prevalent being excitement, with anxiety or trepidation noted on the faces or in the trembling hands of some.

*Two surgeons stand either side of the pig performing an anterolateral thoracotomy. Halfway through the clamshell procedure, the monitor shows that the pig’s heart rate and oxygen saturations have significantly decreased. The veterinarian lifts the surgical drapes and assesses the pig. Once the vital signs normalise, the surgeons can continue. They use a Gigli saw for the first time, passing the wire beneath the sternum and lifting up to begin sawing through the bone. Once the chest is open and the pericardium incised, the beating heart is glistening under the operating lights. The learners are visibly excited; one uses a scalpel to create a stab incision to the right ventricle. There is no significant spurt of blood and they look disappointed. The monitor shows that the pig’s blood pressure is very low*.

*The learners repeatedly practice suturing the heart, frustrated when they get it wrong, elated when they get it right. Steadily, the beating motion has reduced to faint fibrillation. The educator notices the physiological parameters on the monitor and makes eye contact with the veterinarian, as one other learner asks, “is she alive right now?” “She’s in distress,” is the reply, indicating to the educator with their hands that the learners need to start performing cardiac massage. The pig’s physiology suggests that she is close to death. The educator looks around at the other operating tables – “OK, let’s move on and practice on the IVC real quick.”*

The criticality of this aspect of training, indicated a shift more toward pragmatic and interpersonal mediation, especially for the learners who used the appearance and activity of the animal’s organs and physiology to inform their situational awareness and surgical practice, interacting with the veterinarian (surrogate anaesthetist) to get a clinical update on the pig’s status. This activity is highly comparable to human clinical activity. The repeated practice of cardiac suturing, especially with associated emotional reactions – not seen when learners practiced other procedures – may be evidence of pragmatic and reflexive mediation. Learners demonstrated their skills and reflected on their own abilities, allowing them to adapt their practice at the next attempt, taking advantage of the rare opportunity to suture a beating heart.

This vignette further demonstrates the duality of the animal, perceived by different participants at different times. The veterinarians stop the learning activity to assess the pig, as for them, the animal is a patient. The learners treat the animal as both an instrument, disappointed when the heart does not bleed as expected, and later as a patient, noticing the abnormal physiology and demonstrating concern for the animal’s welfare. This duality was also noticed in how the pigs were referred during LTT, either as an object (“it” or “the model”), or using pronouns such as him and her, or names such as Babe or Porky. The latter indicates consideration of the pig as a living being, but the choice of names demonstrates a distinction in the learners’ minds between this animal patient and their human patients.

The educator is responsible for resituating the animal as a model, and returning learning to an epistemic or pragmatic orientation. The importance of keeping the pig alive relates to the paramount aim for learners to practice surgical skills in the presence of bleeding. Their decision to move to operate on the IVC (inferior vena cava, a large thoracoabdominal blood vessel) is motivated for the benefit of the learner and not the patient. The inference is that the learner has to practice this skill before the pig dies, or the opportunity will be missed. This is in stark contrast to clinical practice, where skills are enacted for the benefit of the human patient, and not the learning needs of the surgeon, as this would be considered unethical.

### Success and failure, life and death

*One surgeon creates a large wound in the left ventricle which produces an immediate pressurised gush of bright red blood. A second surgeon calmly puts her finger over the wound. The blood pressure reads 50/25. The circulating staff are asked to deliver a fluid bolus and, in response, they begin to squeeze a saline bag hanging beside the operating table. The surgical team work together to suture the wound, while the patient’s systolic blood pressure jumps up and down. Alarms start sounding and there is suddenly a lot of bleeding from the chest. The heart starts fibrillating… everyone who is not occupied at an operating table has stopped to watch. The defibrillator paddles are handed to one of the learners and a shock is delivered directly to the heart – no change – one learner starts to perform cardiac massage, pumping the exposed heart with his hand. A technician stands near the patient’s head looking anxious, pushing a syringe of medication into one of the tubes connected to the pig. The scene is similar to that of a human cardiac arrest. A second shock is delivered and the monitor shows that the heart has returned to sinus rhythm, although the blood pressure remains low*.

*The educator steps away and removes his theatre gown – “the team were very good, they saved him” – the educational event is complete. A staff member shows them all a thumbs-up sign. The team of surgical learners seem very happy, smiling and cheering as they crowd around the urn for a coffee break*.

*Over the next few minutes, alone on the operating table, the pig’s blood pressure steadily drops to nothing as the heart stops beating. Once dead, the corpse is wheeled away and disposed of*.

Unlike other human patient simulator models, the fact that the pig is alive, means that there is the potential to make mistakes, practicing “technical skills, under the pressure of having consequences”. For all involved, this learning experience is a potential life or death situation. Success means performing all of the prescribed skills and survival of the pig; premature death of the animal, even when all are aware that the pig will die regardless, is considered failure. Evidence of reflexive mediation of learning, if present, was most obvious at this stage, especially when learners were presented with sensory evidence of their failures, triggering them to discuss their actions and what could have been done differently.

Duration of animal survival was variable and difficult to predict, regardless of learner and educators interactions with the animal. Some pigs did not tolerate anaesthesia well and succumbed within an hour of training starting, with one learner stating “but we did very little to him” as if to argue against culpability. The predominant framing of the animal as a simulator model by educators, and some learners, may be responsible for this observed phenomenon.

For a rare few, death of the animal clearly affected them. This tended to be those who framed the animals as patients more consistently within their learning experience. One learner was visibly emotional throughout, explaining that as an “animal-lover” and a vegetarian they did not want to do this, but knew the training would be valuable. Upon premature death of an animal, one surgeon walked away from the operating table; he later reported reflecting on his actions and questioning himself about what he had done wrong, behaviour very similar to that exhibited in clinical practice when a human patient unexpectedly dies, and clear evidence of reflexive mediation of learning. Small signs of concern regarding animal welfare were noted from learners, but mostly evidenced by veterinary staff, likely due to their inherent framing of the animal as a patient. This was most apparent during procedures resulting in major haemorrhage and physiological distress to the animal. Emotion at the point of euthanasia was evidenced with tactile caring movements, such as stroking the pigs’ heads or limbs while the vital signs dissipated. Once it was determined that training was completed, the majority walked away or were instructed to leave by simulation staff, although some learners were required to remove equipment and sew up the carcass. Animals were then disposed of in large containers or wheeled away on gurneys. After death, whether this occurred as a by-product of surgical training or through euthanasia, regardless of how they had been perceived during the simulated learning event, the animals were reduced to expired objects which have no further use and are treated as clinical waste by all involved.

## Discussion

This is the first empirical study exploring the complex sociomaterial nature of learning using a live animal for trauma surgical training. The key finding is the notable duality of the animal as an instrument. Learning is mediated predominantly in epistemic and pragmatic orientation, with increased potential for interpersonal and reflexive mediation when the animal is framed as a patient that needs to be saved, rather than a simulator model.

The concept of simulator ontology has been previously discussed in research regarding human cadavers [33,34]. ‘Ontological transitions’ denote participants conceptualising a human body differently at different times, conceiving them “as not fully people, but also as much more than things.” [33] In LTT, animals are alive, yet there is a stark duality as participants reduce them to models for training, using them akin to an inanimate simulator object early in the course, with a transition toward viewing them as living beings and their ‘patients’ when enacting critical, complex and unfamiliar procedures.

The explicit nature of learning is via a combination of epistemic and pragmatic mediation, using the animal’s physical properties and realistic attributes – by virtue of the fact that it is alive – to practice surgical skills. The teaching observed during LTT is reminiscent of both traditional anatomy teaching, cadaveric simulated learning and surgical apprenticeship teaching within an operating theatre. MacLeod *et al*. have argued that cadaveric-based simulation is unique and irreplaceable “because you simply cannot fake ‘human’” [33]. The LTT experience – especially when training to control major haemorrhage – may be a unique example of surgical training that bridges simulated and clinical learning events, in that you cannot fake ‘being alive’. If learners feel that a simulated learning event is real, it has psychological fidelity. Highly contingent on the animal being alive, the inherent risk of ‘life and death’ is interpreted by the learners as success and failure. Actions have consequences and the outcome of the simulation cannot be wholly controlled. Multimodal mediation is more apparent, and potentially more likely to occur when the animal is framed in the role of patient (arguably, humanised), bringing LTT closer to an authentic clinical experience.

Authenticity is a related, but distinct, concept centred on a learner’s subjective interpretation of the accuracy of the simulated experience [54]. Authenticity emerges as a result of participant interactions, it is difficult to engineer in advance [28]. Features of simulation that learners perceive as highly authentic include behaving and performing as they would in real life, experiencing a sense of urgency to save the patient and uncertainty regarding the outcome [55]. Indeed, “time criticality and bleeding, which creates a real-life event” and invokes an emotional response, have previously been suggested as valued educational features of using a live animal [56]. Authenticity may not necessarily be a desirable characteristic, for example, due to the negative effect of cognitive load on novice learners [57,58].

Surgical learning is obtained through social practices in the clinical learning environment [41,59,60] and during simulation training [47]. Ethnographic work has established that medical students and surgical residents learn through practice, simulated or real, and come to embody perceptions, behaviours and ways of being unique to surgery [42,59]. It is highly likely that reflexive mediated-learning coexists with other forms of mediation throughout LTT as it is instigated by the learners themselves and contingent on their past and present experiences, and intrinsic and extrinsic goals. We believe this will be more likely to occur when the animal is instrumentalised as a patient, and could continue after LTT is completed as learners reflect on the authenticity of their experience.

### Limitations

Our novel choice to use a learning theory previously applied to technologies, does not reflect a typical sociomaterial analysis, however it draws attention to beings that can be considered to have a dual social and material nature. By centralising the importance of the animal within our analysis, our findings are inherently specific to LTT and not generalisable across other types of simulation practices.

Limitations of ethnographic research are well known, and we recognise the lead researcher will have influenced the data generation process and the analysis; reflexivity has been a critical aspect of the research process and documented throughout. We have attempted to produce a rich, credible interpretation of the extensive data in response to our research question, with triangulation through multiple international sites and different methods of data collection, and the inclusion of researchers with alternative backgrounds. A deliberate choice was made not to share fieldnotes, interview transcripts or analytic interpretations with the course convenors and faculty educators, despite the potential benefits of member reflections [61]. Many approached courses were unwilling to allow an external observer to be present. This could be significant, as data generated at those sites may have contributed to alternative interpretations. Challenging aspects of researching this sensitive topic may be reported on in the future.

## Conclusion

Simulation training using a live animal mediates surgical learning in different ways, dependent on whether the animal is framed as an instrument or as a patient. The animal’s ability to bleed and exsanguinate to death creates risk and uncertainty, requiring learners to perform complex skills under pressure of significant consequence. The living nature of the pig affords this opportunity, albeit with associated moral and ethical risk. The pedagogic approach mirrors a clinical learning event, yet by framing the animal as a simulator, mediation of learning is unintentionally limited toward epistemic and pragmatic orientations. Heightening the authenticity by incorporating opportunities for reflexive and interpersonal mediation of learning, made feasible when the animal is framed as a patient, may enrich the learning experience.

Considering the requisite loss of animal life, involvement with LTT might be considered a privilege. We hope our research findings encourage those involved with LTT to consider their justification of live animal use and whether their current practices are reasonable, appropriate, and used to maximal educational advantage.

## References

[1] Gaba DM. The future vision of simulation in health care. BMJ Qual Saf. 2004;13(suppl 1):i2–i10. DOI: 10.1136/qshc.2004.009878 PMC1765792 15465951

[2] Bergmeister KD, Aman M, Kramer A, Schenck TL, Riedl O, Daeschler SC, et al. Simulating surgical skills in animals: Systematic review, costs & acceptance analyses. Front Vet Sci. 2020;7. DOI: 10.3389/fvets.2020.570852 PMC7554573 33195561

[3] Da Luz LT, Nascimento B, Tien H, Kim MJ, Nathens AB, Vlachos S, et al. Current use of live tissue training in trauma: a descriptive systematic review. Can J Surg. 2015;58(3 Suppl 3):S125. DOI: 10.1503/cjs.014114 26100772 PMC4467511

[4] Goolsby C, Branting A, Ausman J, Williams D, Ausman C, David J, et al. Systematic review of live tissue versus simulation education for prehospital trauma providers. Mil Med. 2017;182(9–10):e1824–e33. DOI: 10.7205/MILMED-D-17-00026 28885943

[5] Rubeis G, Steger F. Is live-tissue training ethically justified? An evidence-based ethical analysis. Altern Lab Anim. 2018;46(2):65–71. DOI: 10.1177/026119291804600206 29856644

[6] Swain C, Rickard R, Karlgren K, Helgesson G. Considering the ethics of live tissue training in trauma surgery. J Med Ethics. 2025. DOI: 10.1136/jme-2023-109761 PMC13479244 40044417

[7] Swain C, Stathakarou N, Alzuguren P, Lemarteleur V, Moffatt R, Karlgren K. Trauma surgical simulation: discussing the replacement of live animals used as human patient simulators. Adv Simul. 2024;9(1):7. DOI: 10.1186/s41077-024-00279-2 PMC10860211 38342893

[8] Mahoney A, Reade M, Moffat M. Experiences of medical practitioners in the Australian Defence Force on live tissue trauma training. BMJ Mil Health. 2020. DOI: 10.1136/bmjmilitary-2020-001550 33087539

[9] Schoenherr JR, Hamstra SJ. Beyond Fidelity: Deconstructing the Seductive Simplicity of Fidelity in Simulator-Based Education in the Health Care Professions. Simul Healthc. 2017;12(2):117–23. DOI: 10.1097/SIH.0000000000000226 28704289

[10] Hamstra SJ, Brydges R, Hatala R, Zendejas B, Cook DA. Reconsidering fidelity in simulation-based training. Acad Med. 2014;89(3):387–92. DOI: 10.1097/ACM.0000000000000130 24448038

[11] Tun JK, Alinier G, Tang J, Kneebone RL. Redefining simulation fidelity for healthcare education. Simul Gaming. 2015;46(2):159–74. DOI: 10.1177/1046878115576103

[12] Maran NJ, Glavin RJ. Low-to high-fidelity simulation–a continuum of medical education? Med Educ. 2003;37:22–8. DOI: 10.1046/j.1365-2923.37.s1.9.x 14641635

[13] Clarke SO, Ilgen JS, Regehr G. Fostering Adaptive Expertise Through Simulation. Acad Med. 2023;98(9):994–1001. DOI: 10.1097/ACM.0000000000005257 37094295

[14] Paige JB, Morin KH. Simulation fidelity and cueing: A systematic review of the literature. Clin Simul Nurs. 2013;9(11):e481–e9. DOI: 10.1016/j.ecns.2013.01.001

[15] Choi W, Dyens O, Chan T, Schijven M, Lajoie S, Mancini ME, et al. Engagement and learning in simulation: recommendations of the Simnovate Engaged Learning Domain Group. 2017. DOI: 10.1136/bmjstel-2016-000177

[16] Norman G, Dore K, Grierson L. The minimal relationship between simulation fidelity and transfer of learning. Med Educ. 2012;46(7):636–47. DOI: 10.1111/j.1365-2923.2012.04243.x 22616789

[17] Fenwick T, Dahlgren MA. Towards socio-material approaches in simulation-based education: lessons from complexity theory. Med Educ. 2015;49(4):359–67. DOI: 10.1111/medu.12638 25800296

[18] Stefanidis D, Yonce TC, Green JM, Coker AP. Cadavers versus pigs: which are better for procedural training of surgery residents outside the OR? Surgery. 2013;154(1):34–7. DOI: 10.1016/j.surg.2013.05.001 23809483

[19] Izawa Y, Hishikawa S, Muronoi T, Yamashita K, Maruyama H, Suzukawa M, et al. Ex-vivo and live animal models are equally effective training for the management of a penetrating cardiac injury. World J Emerg Surg. 2016;11(1):45. DOI: 10.1186/s13017-016-0104-3 27588035 PMC5007845

[20] King DR, Patel MB, Feinstein AJ, Earle SA, Topp RF, Proctor KG. Simulation training for a mass casualty incident: two-year experience at the Army Trauma Training Center. J Trauma Acute Care Surg. 2006;61(4):943–8. DOI: 10.1097/01.ta.0000233670.97515.3a 17033566

[21] Gaarder C, Naess PA, Buanes T, Pillgram-Larsen J. Advanced surgical trauma care training with a live porcine model. Injury. 2004;36(6):718–24. DOI: 10.1016/j.injury.2004.12.024 15910823

[22] Tugnoli G, Ribaldi S, Calderale SM. Learning on animal models: a 16-year experience with the theoretical-practical course on surgery of polytrauma. Ann Ital Chir. 2019;90:379–81. 31815734

[23] Back DA, Waldmann K, Hauer T, Huschitt N, Bowyer MW, Wesemann U, et al. Concept and evaluation of the German War Surgery Course – Einsatzchirurgie-Kurs der Bundeswehr. J R Army Med Corps. 2017;163(3):206–10. DOI: 10.1136/jramc-2016-000706 27909067

[24] Tugnoli G, Ribaldi S, Casali M, Calderale SM, Coletti M, Alifano M, et al. Initial evaluation of the” Trauma surgery course”. World J Emerg Surg. 2006;1(1):1–5. DOI: 10.1186/1749-7922-1-5 16759403 PMC1459266

[25] Fenwick T. Sociomateriality and learning: A critical approach. The SAGE Handbook of Learning. 2015:83–93. DOI: 10.4135/9781473915213.n8

[26] Paavola S, Hakkarainen K. The knowledge creation metaphor–An emergent epistemological approach to learning. Sci Educ. 2005;14:535–57. DOI: 10.1007/s11191-004-5157-0

[27] Kaufmann D. Teaching and Learning in Medical Education: How Theory can Inform Practice. In: Swanwick T, Forrest K, O’Brien BC, editors. Understanding Medical Education: Evidence, Theory and Practice. 3rd ed. Oxford, UK: Wiley Blackwell; 2019. pp. 37–69. DOI: 10.1002/9781119373780.ch4

[28] Rystedt H, Sjöblom B. Realism, authenticity, and learning in healthcare simulations: rules of relevance and irrelevance as interactive achievements. Instr Sci. 2012;40(5):785–98. DOI: 10.1007/s11251-012-9213-x

[29] Handeland JA, Prinz A, Ekra EMR, Fossum M. The sense of a patient: An ethnographic multi-site field study exploring the influence of manikins on nursing students’ learning. Int J Educ Res Open. 2022;3:100110. DOI: 10.1016/j.ijedro.2021.100110

[30] Ahn S, Rimpiläinen S. Maintaining Sofia–or how to reach the intended learning outcomes during a medical simulation training. Int J Learn Tech. 2018;13(2):115–29. DOI: 10.1504/IJLT.2018.092095

[31] Hindmarsh J, Hyland L, Banerjee A. Work to make simulation work: ‘Realism’, instructional correction and the body in training. Discourse Stud. 2014;16(2):247–69. DOI: 10.1177/1461445613514670

[32] Johnson E. Surgical simulators and simulated surgeons: Reconstituting medical practice and practitioners in simulations. Soc Stud Sci. 2007;37(4):585–608. DOI: 10.1177/0306312706072179 18175617

[33] MacLeod A, Luong V, Cameron P, Kovacs G, Fredeen M, Patrick L, et al. The Lifecycle of a Clinical Cadaver: A Practice-Based Ethnography. Teach Learn Med. 2022;34(5):556–72. DOI: 10.1080/10401334.2022.2092111 35770381

[34] MacLeod A, Cameron P, Luong V, Kovacs G, Patrick L, Fredeen M, et al. Negotiating humanity: an ethnography of cadaver-based simulation. Adv Health Sci Educ. 2023;28(1):181–203. DOI: 10.1007/s10459-022-10152-4 PMC9395868 35994215

[35] Rabardel P, editor. Instrument mediated activity in situations. People and Computers XV—Interaction without Frontiers: Joint Proceedings of HCI 2001 and IHM 2001; 2001: Springer. DOI: 10.1007/978-1-4471-0353-0_2

[36] Vygotsky LS. The instrumental method in psychology. The concept of activity in Soviet psychology. 1981;2(3):135–43.

[37] Rabardel P, Beguin P. Instrument mediated activity: from subject development to anthropocentric design. Theor Issues Ergon Sci. 2005;6(5):429–61. DOI: 10.1080/14639220500078179

[38] Lonchamp J. An instrumental perspective on CSCL systems. Int J Comput Support Collab Learn. 2012;7:211–37. DOI: 10.1007/s11412-012-9141-4

[39] Andreassen P, Christensen MK, Møller JE. Focused ethnography as an approach in medical education research. Med Educ. 2020;54(4):296–302. DOI: 10.1111/medu.14045 31850537

[40] Rashid M, Hodgson CS, Luig T. Ten tips for conducting focused ethnography in medical education research. Med Educ Online. 2019;24(1):1624133. DOI: 10.1080/10872981.2019.1624133 31146655 PMC6567138

[41] Prentice R. Surgical teamwork and the pragmatic ethics of the outcome. Med Anthropol. 2021;40(4):361–74. DOI: 10.1080/01459740.2021.1892666 33734924

[42] Prentice R. Bodies in formation: An ethnography of anatomy and surgery education. Duke University Press; 2013. DOI: 10.2307/j.ctv111jhxr

[43] Katz P. Ritual in the operating room. Ethnology. 1981;20(4):335–50. DOI: 10.2307/3773355

[44] Katz P. The scalpel’s edge: The culture of surgeons. 1999.

[45] Cassell J. Expected miracles: Surgeons at work. Temple University Press; 2010.

[46] Hirschauer S. The manufacture of bodies in surgery. Soc Stud Sci. 1991;21(2):279–319. DOI: 10.1177/030631291021002005

[47] Cleland J, Walker KG, Gale M, Nicol LG. Simulation-based education: understanding the socio-cultural complexity of a surgical training ‘boot camp’. Med Educ. 2016;50(8):829–41. DOI: 10.1111/medu.13064 27402043

[48] Davies CA. Reflexive ethnography: A guide to researching selves and others. Routledge; 2012.

[49] Geertz C. Thick description: Toward an interpretive theory of culture. The cultural geography reader. Routledge; 2008. pp. 41–51.

[50] Emerson RM, Fretz RI, Shaw LL. Writing ethnographic fieldnotes. University of Chicago press; 2011. DOI: 10.7208/chicago/9780226206868.001.0001

[51] Polkinghorne DE. Narrative configuration in qualitative analysis. Int J Qual Stud Educ. 1995;8(1):5–23. DOI: 10.1080/0951839950080103

[52] Mattingly C. Healing dramas and clinical plots: The narrative structure of experience. Cambridge University Press; 1998. DOI: 10.1017/CBO9781139167017 PMC1115609 10221969

[53] Russell WMS, Burch RL. The principles of humane experimental technique. London, UK: Methuen; 1959.

[54] Corves C, Stadler M, Fischer MR. Perceived authenticity across three forms of educational simulations—the role of interactant representation, task alignment, and continuity of simulation. Eur J Psych Educ. 2024:1–23. DOI: 10.1007/s10212-024-00826-5

[55] Lavoie P, Deschênes M-F, Nolin R, Bélisle M, Garneau AB, Boyer L, et al. Beyond technology: A scoping review of features that promote fidelity and authenticity in simulation-based health professional education. Clin Simul Nurs. 2020;42:22–41. DOI: 10.1016/j.ecns.2020.02.001

[56] Swain CS, Cohen HML, Helgesson G, Rickard RF, Karlgren K. A Systematic Review of Live Animal Use as a Simulation Modality (“Live Tissue Training”) in the Emergency Management of Trauma. J Surg Educ. 2023;80(9):1320–39. DOI: 10.1016/j.jsurg.2023.06.018 37516576

[57] Howie EE, Dharanikota H, Gunn E, Ambler O, Dias R, Wigmore SJ, et al. Cognitive Load Management: An Invaluable Tool for Safe and Effective Surgical Training. J Surg Educ. 2023. DOI: 10.1016/j.jsurg.2022.12.010 36669990

[58] Frederiksen JG, Sørensen SMD, Konge L, Svendsen MBS, Nobel-Jørgensen M, Bjerrum F, et al. Cognitive load and performance in immersive virtual reality versus conventional virtual reality simulation training of laparoscopic surgery: a randomized trial. Surg Endosc. 2020;34:1244–52. DOI: 10.1007/s00464-019-06887-8 31172325

[59] Prentice R. Drilling surgeons: The social lessons of embodied surgical learning. Sci Technol Human Values. 2007;32(5):534–53. DOI: 10.1177/0895904805303201

[60] Jensen RD, Seyer-Hansen M, Cristancho SM, Christensen MK. Being a surgeon or doing surgery? A qualitative study of learning in the operating room. Med Educ. 2018;52(8):861–76. DOI: 10.1111/medu.13619 29992693

[61] Tracy SJ. Qualitative quality: Eight “big-tent” criteria for excellent qualitative research. Qual Inq. 2010;16(10):837–51. DOI: 10.1177/1077800410383121

